# Genome-Wide Identification of Reference Genes for Reverse-Transcription Quantitative PCR in Goat Rumen

**DOI:** 10.3390/ani11113137

**Published:** 2021-11-02

**Authors:** Juan Zhao, Cheng Wang, Lin Zhang, Aiai Lei, Linjie Wang, Lili Niu, Siyuan Zhan, Jiazhong Guo, Jiaxue Cao, Li Li, Hongping Zhang, Tao Zhong

**Affiliations:** Farm Animal Genetic Resources Exploration and Innovation Key Laboratory of Sichuan Province, College of Animal Science and Technology, Sichuan Agricultural University, Chengdu 611130, China; 2020202035@stu.sicau.edu.cn (J.Z.); wxx79595@163.com (C.W.); zl09031111@163.com (L.Z.); sicaulei@163.com (A.L.); wanglinjie@sicau.edu.cn (L.W.); niulili@sicau.edu.cn (L.N.); siyuanzhan@sicau.edu.cn (S.Z.); jiazhong.guo@sicau.edu.cn (J.G.); jiaxuecao@sicau.edu.cn (J.C.); lily@sicau.edu.cn (L.L.); zhp@sicau.edu.cn (H.Z.)

**Keywords:** caprine, rumen, genome-wide, reference gene, RT-qPCR

## Abstract

**Simple Summary:**

The rumen plays an essential role as a digestive organ and serves as the primary site of energy substrate absorption for the productive ruminants. Understanding gene expression profiles is necessary to explore the intrinsic regulatory mechanisms of rumen development in goats. The selection of suitable reference genes (RGs) was the primary assay before the real-time quantitative PCR (RT-qPCR). We identified sixteen genome-wide candidate RGs for normalization of gene expression assessments in goat rumen tissues. We demonstrate that the RGs selected (*RPS4X* and *RPS6*) were more stably expressed than the commonly used HKGs (*ACTB* and *GAPDH*) in goat rumen tissues, suggesting that the ribosomal protein gene family may be another source for the RG pool.

**Abstract:**

As the largest chamber of the ruminant stomach, the rumen not only serves as the principal absorptive surface and nutrient transport pathway from the lumen into the animal, but also plays an important short-chain fatty acid (SCFA) metabolic role in addition to protective functions. Accurate characterization of the gene expression profiles of genes of interest is essential to the exploration of the intrinsic regulatory mechanisms of rumen development in goats. Thus, the selection of suitable reference genes (RGs) is an important prerequisite for real-time quantitative PCR (RT-qPCR). In the present study, 16 candidate RGs were identified from our previous transcriptome sequencing of caprine rumen tissues. The quantitative expressions of the candidate RGs were measured using the RT-qPCR method, and the expression stability of the RGs was assessed using the geNorm, NormFinder, and BestKeeper programs. GeNorm analysis showed that the M values were less than 0.5 for all the RGs except *GAPT4*, indicating that they were stably expressed in the rumen tissues throughout development. *RPS4X* and *RPS6* were the two most stable RGs. Furthermore, the expressions of two randomly selected target genes (*IGF*1 and *TOP2A*), normalized by the selected most stable RGs (*RPS4X* and *RPS6*), were consistent with the results of RNA sequencing, while the use of *GAPDH* and *ACTB* as RGs resulted in altered profiles. Overall, *RPS4X* and *RPS6* showed the highest expression stability and the lowest coefficients of variation, and could be used as the optimal reference combination for quantifying gene expression in rumen tissues via RT-qPCR analysis.

## 1. Introduction

The reverse-transcription quantitative real-time polymerase chain reaction (RT-qPCR) is a well-established method for quantifying mRNA expression, in addition to northern blotting, in situ hybridization, and ribonuclease protection assay [1,2]. Compared with other technologies, RT-qPCR has the advantages of rapidity, accuracy, high sensitivity, and good reproducibility. Thus, it is extensively used to compare mRNA transcription in different biological samples, tissues, or cells [3,4]. The accuracy of RT-qPCR is primarily dependent on the selection of suitable RGs [5], which act as an internal control for the normalization of the expression of target genes. Perfect RGs should be stably expressed in diverse individuals, tissues, and cells without spatiotemporal differences, even under different experimental treatments [6]. However, several studies have found that the stabilities of RGs turned out to be species-, tissue-, or cell-specific [7,8]. Lee and colleagues revealed considerable variability of 12 commonly used RGs within and across microarray datasets, including in different mammalian cell contexts [9]. Therefore, the screening of suitable RGs is a vital prerequisite to quantifying the expression profiles of target genes.

Commonly used RGs such as *GAPDH*, *ACTB*, and ribosomal genes are considered to be expressed continuously and stably during a cell’s life activities, and are called housekeeping genes (HKGs) [1,6,10]. Sometimes, expressions of HKGs are not very stable or otherwise do not meet the criteria for a suitable internal control [11]. For instance, *GAPDH* and *ACTB* were found to not be expressed stably in the skeletal muscle of growing mice, although they were used as the single RG in more than 90% of the quantification studies [1]. More than one RG shoud be used to normalize the expressions of target genes. More candidate novel HKGs, predominantly ribosomal protein genes, were identified by a meta-analysis of more than 13,000 samples in humans [12]. The phenomenon was reported that a more significant expression error was commonly associated with increased noise and inability to detect minor differences [13]. Besides RNA yield, quality, and reverse transcription efficiency [4,7,14], it is also necessary to apply a data normalization strategy to eliminate the variations caused by technology or experiment. At present, the geNorm, NormFinder, and BestKeeper software tools are primarily used to evaluate the expression stability of candidate RGs, and to identify the minimum number of RGs needed under diverse experimental conditions [15,16].

Extensive studies have been conducted to explore the mechanisms of digestion and absorption of nongrain feed in ruminants in order to improve the feed conversion efficiency, especially for pigs and chickens [17,18,19]. As a unique digestive organ, the rumen is the main chamber for the absorption and transportation of nutrients, providing approximately 70% of energy for ruminants [20]. Numerous studies have pointed out the main mechanisms related to the absorption and transport of nutrients, such as passive diffusion [21], acid anion exchange [22], proton-coupled volatile fatty acid (VFA) transport [23], and electrically mediated VFA transport [20]. Many candidate genes and noncoding RNAs have been investigated to explore their functions during rumen development [24,25,26,27,28]. Therefore, the selection and validation of RGs are necessary prior to assessing the expression levels of candidate genes without bias.

In the literature, a limited number of candidate RGs (*ACTB*, *UXT*, *DBNDD2*, *RPS9*, *DDX54*, and *HMBS*) have been validated to be expressed stably in the rumen epithelial tissues of cattle [29]. Recently, more novel RGs were identified and validated to be more stable than the traditional HKG in the skin tissues of Chinese indigenous goats based on high-thoughput sequencing technology [30]. Thus, we conducted a genome-wide search for RGs in goat rumen tissues using our previous RNA-sequencing data. In the present study, 16 candidate RGs were selected and quantified for their expression levels using RT-qPCR. Subsequently, the stability and applicability of these RGs were evaluated using geNorm, NormFinder, and BestKeeper procedures to determine the optimal RGs in goat rumen tissues.

## 2. Materials and Methods

### 2.1. Animals, RNA Isolation, and cDNA Synthesis

In this study, 12 Chengdu Brown goats [31] (six 2-month-old goats and six 1-year-old goats) were randomly selected in the Chengdu Xilingxue Agricultural Development Co., Ltd. (Sichuan, China). All the goats were reared according to the local standards of Sichuan Province (DB51/T654-2007). After slaughter, rumen samples were collected rapidly, placed in 2 mL cryogenic tubes without RNA enzyme, and immediately frozen in liquid nitrogen.

Total RNA was extracted using the Animal Total RNA Isolation Kit (FOREGENE, Chengdu, China) according to the methods described by the manufacturers. The purity and integrity of RNA were evaluated using 1.0% (*w/v*) agarose gel electrophoresis and an Agilent 2100 Bioanalyzer System (Agilent, Santa Clara, CA, USA). The concentration of RNA was measured using the NanoDrop 2000 spectrophotometer (Thermo Scientific, Waltham, MA, USA). The cDNA was synthesized using the Reverse Transcription Kit (Takara, Dalian, China) following the producer’s protocol. The synthesized cDNA was stored at −20 °C.

### 2.2. Genome-Wide Selection of the Candidate RGs

An appropriate RG should not only be expressed stably between tissues and biological states, but also have a higher level than the background [14]. Based on our RNA-sequencing data of 12 goat rumen tissues (PRJNA720177), the candidate genes were preliminarily selected according to the three parameters (fragments per kilobase of exon model per million mapped reads (FPKM), FDR, and CV). The FPKM value was greater than 100 and the absolute fold change of log2-converted absolute FPKM less than 1, the false discovery rate (FDR) was less than 1, and the coefficient of variation (CV) was less than 0.2. Subsequently, eight genes involved in the ribosome, aminoacyl-tRNA biosynthesis, and HIF-1 signaling pathways were selected as the candidate RGs (Table S1). In addition, another eight previously reported RGs were jointly analyzed in this study, including the two most used RGs (*ACTB* and *GAPDH*).

### 2.3. Quantitative Real-Time PCR (RT-qPCR) and Amplification Efficiency

The qPCR primer pairs of the tested RGs were designed using the Primer Premier 5.0 software (PREMIER Biosoft, Palo Alto, CA, USA) (Table 1). The primers were synthesized by Sangon Biotech (Shanghai) Co., Ltd. The RT-qPCR was performed in a 10 μL system, including 5 μL SYBR Green Real-Time PCR Master Mix (Takara, Dalian, China), 0.4 µL each of forward and reverse primer (10 µM), 3.4 µL RNase-free ddH_2_O, and 0.8 μL cDNA. The cycling conditions were as follows: 3 min at 94 °C for enzyme activation, followed by 35 cycles of 30 s at 95 °C and 30 s at 56.9 °C or 59.4 °C for annealing, 1 min at 72 °C, and final extension for 7 min.

To evaluate the specificity of the designed primers, melting curve analysis and agarose gel electrophoresis were performed to detect the nonspecific product of each primer pair. The standard curve of the qPCR was established using the gradient diluted cDNA. The correlation coefficient and amplification efficiency were calculated using the CFX ManagerTM Software (Bio-Rad, Hercules, CA, USA). The calculation equation for primer amplification efficiency was as follows: E% = (10^(−1/slope)^ − 1) × 100%.

### 2.4. The Expression Stability of the Candidate RGs in Rumens

To assess the optimal set of candidate RGs in the rumen tissues, the stability of each RG was analyzed using the geNorm (PrimerDesign, Southampton, Hample, England), NormFinder (Aarhus University Hospital, Aarhus, Denmark), and BestKeeper (Microsoft, Redmond, Washington, DC, USA). The relative expression quantity (Q) of each candidate internal RG was calculated as follows: Q = 2^−ΔCt^, ΔCt = Ct _(sample)_ − Ct _(minimum)_, where Ct _(sample)_ was the Ct value of a factor in each sample and Ct _(minimum)_ was the minimum Ct value of this gene in all samples.

The gene expression stability (M value) was calculated using geNorm by comparing a particular gene with other RGs [2,32]. The lower the value of M, the better the stability of the RG [33]. By gradually eliminating the RGs with the largest M values, all candidate RGs were ranked and the optimal number of RGs was selected. NormFinder was used to calculate stability values for candidate RGs by analyzing their intragroup and intergroup variation [6,34]. The RG with the lowest stability value was considered to be the most stable gene [35]. BestKeeper was used to analyze the expression stability of candidate RGs by calculating the coefficient of variance (CV) and the standard deviation (SD) based on the raw Ct values from RT-qPCR [6]. The RGs with the highest stability had the lowest values of CV and SD, while harboring the highest value of correlation coefficient (r). In addition, two target functional genes (*TOP2A* and *IGF1*) were chosen to validate the effects of the different RGs on their expression. All samples were evaluated in triplicate, and their relative expression levels were estimated using the 2^−ΔΔCt^ method.

### 2.5. Statistical Analyses

Statistical analyses were conducted using SPSS 20.0 software (IBM, Armonk, NY, USA). All data are expressed as mean ± SEM; data were compared by one-way ANOVA and Duncan’s new multiple range tests, and P-values lower than 0.05 were considered statistically significant. Statistical analysis was performed using the Graph Pad Prism 6.01 program (GraphPad, San Diego, CA, USA).

## 3. Results

### 3.1. The Selection of RGs in Goat Rumen Tissues

According to the values of FPKM and coefficient of variation, a total of 71 candidate RGs were obtained (Supplementary Table S1). Subsequently, KEGG enrichment analysis showed that most of the 71 candidate RGs were involved in the ribosome, aminoacyl-tRNA biosynthesis, and HIF-1 signaling pathways (Table S1). Thus, we selected eight representative RGs (*RPS20*, *RPL7*, *RPL3*, *RPS26*, *RPS4X*, *RPS6*, *KARS*, and *RPS27A*) to evaluate their expression stability. In addition, five RGs (*GPAT4*, *HMBS*, *CALM2*, *DYNLL1*, and *FTH1*) were selected based on previous studies on these rumen tissues. We also used three traditional RGs, including *GAPDH*, *YWHAZ*, and *ACTB* genes.

### 3.2. RNA Purity, Primer Verification, and Amplification Efficiency

The OD260/280 ratios of the RNA samples ranged from 1.81 to 2.12 and their RNA integrity number (RIN) values ranged from 8.1 to 9.1 (Supplementary Table S2), indicating that the isolated RNA was of high quality and suitable for subsequent analysis. As represented in Supplementary Figures S1 and S2, a single band with the expected size was amplified and visualized on the agarose gel, and a single peak was detected in the melting curve of each primer pair, indicating that all the 16 primer pairs could amplify the target fragment with high specificity. In addition, the standard curves of the tested RGs showed better linear relationships, with their efficiency ranging from 92.3% to 107.0%, and all of the correlation coefficients were higher than 0.98 (Table 1, Supplementary Figure S3).

### 3.3. Gene Expression Dispersion Analysis

**Figure 1:**
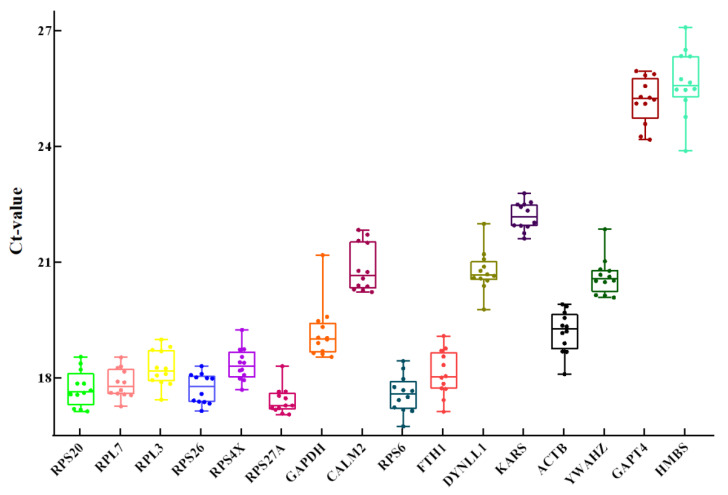
Range and dispersion of Ct values of candidate RGs obtained from all cDNA samples. The ends of each box represent 25% and 75% of the quartile range. The line across the box represents the median value.

The average Ct values of all cDNA samples from candidate internal RGs ranged from 16.74 to 27.08 (Figure 1). The lower the dispersion of the Ct value, the higher the stability of the gene. Among the candidate genes tested, the transcript abundance was highest for *RPS6* (average FPKM, 959.165) and the lowest for *HMBS* (average FPKM, 6.141). In brief, the lowest Ct dispersion was observed for *RPS27A* (17.05 ≤ Ct ≤ 18.30), followed by *KARS*, *RPL7*, *RPS26*, and *RPS4X*, while the highest variation was noted for *HMBS* (23.88 ≤ Ct ≤ 27.08). These results indicated that the most stable gene in terms of mRNA expression levels was *RPS27A*, while the least stable one was *HMBS*.

### 3.4. Expression Stability of the RGs Assessed via geNorm Analysis

**Figure 2:**
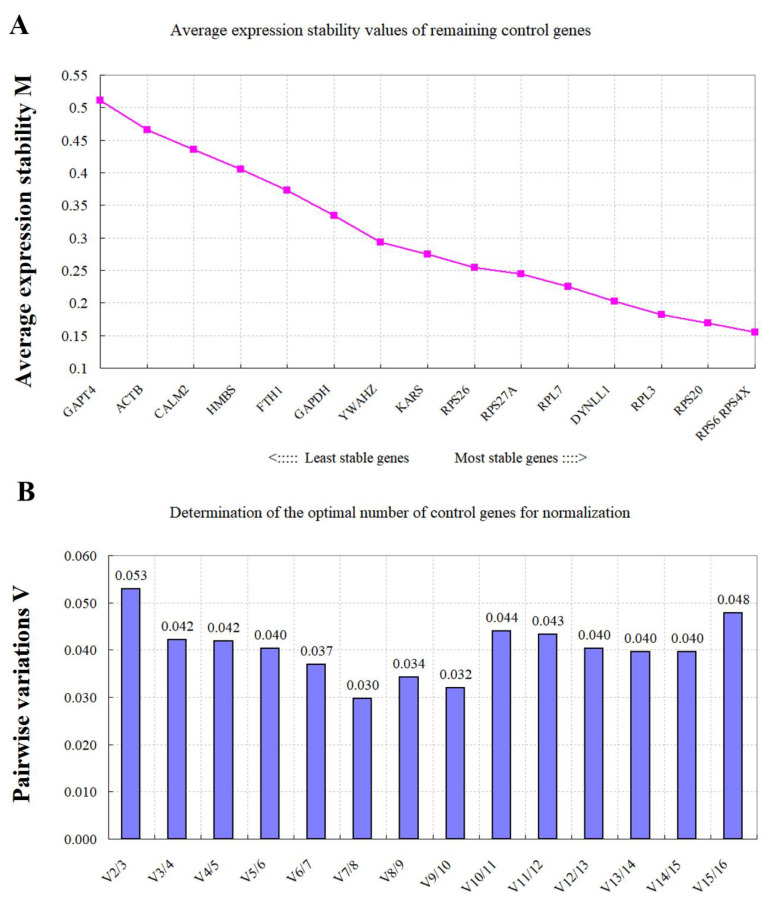
geNorm analysis of the expression stability of the 16 selected RGs. (**A**) The average expression stability measure (M) and ranking of the candidate RGs were calculated using geNorm. A lower M value indicated a more stable expression. (**B**) Determination of the optimal number of RGs for normalization by the pairwise variation (V) as assessed using geNorm. The optimal number of RGs was indicated by V values below 0.15 [34].

As shown in Figure 2, most of the commonly used RGs displayed acceptably low variability (M < 0.5). *RPS4X*, *RPS6*, *RPS20*, and *RPL3* were the most stable genes with the lowest M values (M < 0.2). Similarly, *GAPT4*, *ACTB*, *CALM2*, *HMBS*, *FTH1*, and *GAPDH* were the least stable genes with the highest M value (Figure 2A). In addition, geNorm was used to calculate the number of optimal RGs to derive the normalization factor (NF). The optimal number of RGs recommended in this experiment by the pairwise-variation analysis (Figure 2B) was two, indicating that the most stable RGs (*RPS4X* and *RPS6*) would be sufficient to normalize gene expression among all the rumen samples used in this study.

### 3.5. Expression Stability of the RGs Assessed via NormFinder Analysis

**Figure 3:**
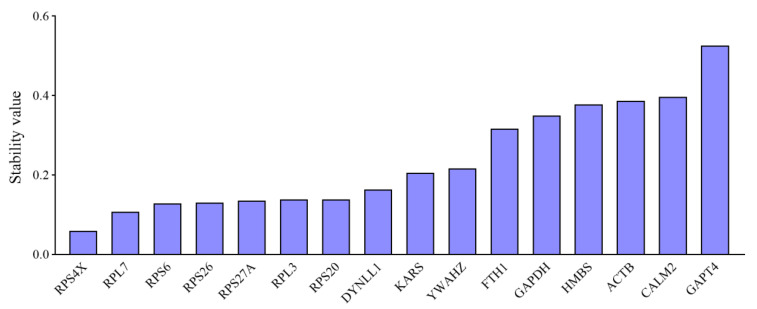
Expression stability of RGs according to NormFinder analysis.

As analyzed using the geNorm method, the RGs with the lowest M values were considered to be the most stable. Based on the results of NormFinder analysis, the stabilities of 16 candidate RGs were ranked (Figure 3). *RPS4X* was the most stable gene, with a stability value less than 0.1, followed by *RPL7*, *RPS6*, *RPS26*, *RPS27A*, *RPL3*, *RPS20*, and *DYNLL1*, while *GAPT4*, *ACTB*, *CALM2*, *HMBS*, *FTH1*, and *GAPDH* were the six least stable RGs.

### 3.6. Expression Stability of the RGs Assessed via Bestkeeper Analysis

The most stable RGs were selected based on the lowest coefficient of variance and standard deviation. BestKeeper can only calculate the RGs within 10 numbers. Therefore, the six least stable RGs (*GAPT4*, *ACTB*, *CALM2*, *HMBS*, *FTH1*, and *GAPDH*) were excluded by combining the results of GeNorm and NormFinder. In the 10 RGs that remained, *RPS4X* and *RPS6* were stably expressed in all the samples (Table 2).

### 3.7. Normalizing the Expression Profiles of Target Genes Using the Target RGs

**Figure 4:**
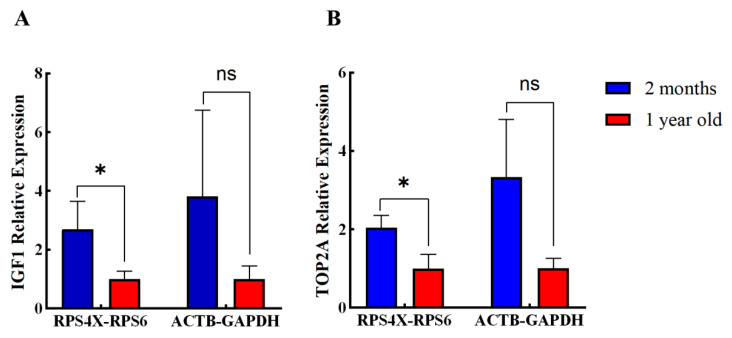
The relative expressions of *IGF1* (**A**) and *TOP2A* (**B**) normalized to the different RGs combinations (the most stable *RPS4X* and *RPS6*, and the least stable *GAPDH* and *ACTB*) using rumen tissues sampled at two developmental stages. * *p* < 0.05. “ns” means no significant difference between the two groups.

To further verify the selection of candidate RGs, the most stable RGs (*RPS4X* and *RPS6*) or the least stable RGs (*GAPDH* and *ACTB*) were used to standardize the same target. When *IGF1* and *TOP2A* were normalized using *RPS4X* and *RPS6* as internal RGs, there was a significant difference between the expression levels of *IGF1* and *TOP2A* in the rumen tissues from 2-month-old and 1-year-old goats (Figure 4). In contrast, when the data were normalized to *GAPDH* and *ACTB*, there was no significant difference in the expression levels of *IGF1* and *TOP2A*.

## 4. Discussion

A large proportion of RGs were selected according to previous literature, of which many were housekeeping genes (HKGs) [7,36,37]. Moreover, it is widely accepted that the expression levels of HKGs are not stable in some species or tissues, and cannot meet the criteria of RG normalization [38,39,40]. With the development of high-throughput sequencing technology, the selection strategy of RGs based on genome-wide analysis has become more powerful. Seven novel RGs, including *RPS4X*, were verified to be more stable than the traditional RGs (*ACTB* and *GAPDH*) in the luteal corpus of Holstein cattle during early gestation and luteolysis [41]. In the skin tissues from Chinese indigenous goats, a RG set consisting of *NCBP3*, *SDHA*, and *PTPRA* were filtered out using transcriptome data and displayed more stable expression than previously used HKGs [30]. Similarly, our results based on genome-wide data analysis also show that a new set of candidate genes, *RPS4X* and *RPS6*, are more stable in expression than *ACTB* and *GAPDH*, suggesting that the traditional RGs may not be ideal RGs, especially for the gene expression assay used in goat rumen tissues.

In ruminants, the digestion and absorption of nutrients occurs mainly in the rumen and small intestine. Carbohydrates are degraded into volatile fatty acids and absorbed by the rumen epithelial cells [29]. Many studies have reported related gene expression profiles during rumen development in goats [24,28,42,43,44]. To date, few studies have assessed the applicability of HKG standardization in this tissue, and the identification of RGs has not been based on genome-wide identification. Therefore, we conducted a genome-wide analysis of RGs in rumen tissues of goats using our previous RNA sequencing data (rumen tissuess sampled at four developmental stages) and identified a total of 71 potentially stable genes in the study. Eight putative RGs were selected through functional enrichment analysis, all of which belonged to the ribosomal protein gene family except *KARS*. *KARS* is a member of the RAS protein family, which is mainly involved in cell proliferation [45,46]. *KARS* encodes both cytoplasmic and mitochondrial lysyl-tRNA synthetase, a moonlighting protein that has a typical function in protein synthesis [47,48]. In addition, eight genes were selected from the previously reported literature [29,49], including two traditional RGs (*ACTB* and *GAPDH*). The stability of these 16 candidate RGs was analyzed by RT-qPCR, and 10 genes were identified to be more stable in rumen development than *ACTB* and *GAPDH*. After a comprehensive analysis of the stability of these candidate RGs and the normalization effect of target genes, we concluded that *RPS4X* and *RPS6* was the most stable combination of RGs during rumen development, suggesting that the ribosomal protein gene family might be another RG source.

Ribosomal proteins play important housekeeping roles in ribosomal biogenesis and protein production and are essential for cell growth, proliferation, differentiation, and development in animals [50,51]. Some ribosomal proteins have been shown to play critical roles in tightly coordinating *p53* signaling with ribosomal biogenesis. *RPS27A* and *RPS20*, for example, interact with the central acid domain of *MDM2* and inhibit *MDM2*-mediated *p53* ubiquitination, mediating *p53* activation and cell cycle arrest [52,53]. In addition, mutations in the ribosomal protein genes have been found to be involved in cancers such as endometrial cancer (*RPL22*), chronic lymphoblastic leukemia (*RPS15*), colorectal cancer (*RPS20*), and glioma (*RPL5*) [54]. Phosphorylation of *RPS6* attenuates DNA damage and p53-mediated tumor suppression during pancreatic cancer development [55]. *RPS4X* and *RPS4Y* encode different subtypes of the *RPS4*. *RPS4*, a cysteine protease [56], has been poorly studied. It has been suggested that loss of *RPS4* expression may be associated with the development of Turner syndrome (congenital ovarian hypoplasia) [57]. We demonstrate that the ribosomal protein genes (*RPS4X* and *RPS6*) were expressed stably during rumen development in goats. Thus, it can be seen that ribosomal protein-coding genes play a critical role in cell life activities, animal growth, and development, and may be an important source of housekeeping genes.

## 5. Conclusions

To verify normalized gene expression in rumen tissues of goats, we identified 16 candidate RGs using the three tools (geNorm, NormFinder, and BestKeeper) based on transcriptome sequencing. We demonstrated that the expression of the RGs (*RPS4X* and *RPS6*) selected are more stable than the commonly used HKGs (*ACTB* and *GAPDH*) in goat rumen tissues collected at different developmental stages, suggesting that the ribosomal protein gene family may be another source of RGs. This study provides a new idea for screening internal reference genes.

## Figures and Tables

**Table 1:** The primer sequence information, standard curve amplification efficiency, and R^2^ values used in this study.

Gene	Accession No.	Sequences (5′-3′)	Tm (°C)	Size (bp)	Slope	Efficiency (%)	R^2^
*RPS20*	XM_013969227.2	F: ATCAGAGGCGCGAAGGAAAA	56.9	158	−3.421	96.0%	1.000
		R: TGCAGGTCAATGAGTCGCTT					
*RPL7*	XM_005689063.3	F: ACTTCCTGTGGCCCTTTAA	56.9	103	−3.489	93.5%	0.993
		R: ATCTGGTCTTCCCTGTTGC					
*RPL3*	XM_005681086.3	F: CTGACAAGAGCATCAACCC	56.9	209	−3.472	94.1%	0.999
		R: GAAGCGACCATGACCAAAT					
*RPS26*	XM_013963957.2	F: GAACAACGGTCGTGCCAAAA	56.9	171	−3.431	95.6%	0.993
		R: ACGTAGGCGTCGAAAACACT					
*RPS4X*	XM_005700650.3	F: TACTTGGCCTCCTCAGGTGT	59.4	223	−3.178	106.4%	0.999
		R: TACTTGGCCTCCTCAGGTGT					
*RPS27A*	XM_005686612.3	F: TCTAGTGTTGAGACTTCGTGGTG	59.4	183	−3.523	92.3%	0.997
		R: CCAGCACCACATTCATCTGAGG					
*GAPDH*	XM_005680968.3	F: GCAAGTTCCACGGCACAG	59.4	249	−3.398	96.9%	1.000
		R: GGTTCACGCCCATCACAA					
*CALM2*	XM_005686574.3	F: AGAAGCATTCCGTGTGTTT	56.9	159	−3.495	93.3%	0.995
		R: TCATAGTTTACTTGACCAT					
*RPS6*	XM_005683632.3	F: GGACTGGAGAGAGAAAGCG	59.4	211	−3.324	99.9%	0.996
		R: ACAACATACTGGCGGACAT					
*FTH1*	NM_001285609.1	F: GCTTGGAAAGAAGTGTGAA	56.9	153	−3.364	98.3%	0.992
		R: GCAGGTTGGTTATGTGGTC					
*DYNLL1*	XM_018061128.1	F: GCCGTAATCAAGAATGCCGA	56.9	172	−3.285	101.6%	1.000
		R: CGAAGTTCCTCCCCACGATG					
*KARS*	XM_005691813.3	F: AATCACAGTGCTGATGATGGCA	59.4	94	−3.256	102.8%	0.999
		R: TCAGCTGGTGGATTGCTTGG					
*ACTB*	XM_018039831.1	F: CCTGCGGCATTCACGAAACTAC	59.4	87	−3.223	104.3%	0.997
		R: ACAGCACCGTGTTGGCGTAGAG					
*YWHAZ*	XM_018058314.1	F: ACTACTATCGCTACTTGGCTGAG	59.4	84	−3.264	102.5%	0.998
		R: CTTCTTGTTATGCTTGCTGTGA					
*GPAT4*	XM_018041983.1	F: GGAGTCTCCTTTGGTATCCG	56.9	128	−3.165	107.0%	0.992
		R: CCATTGGTGTAGGGCTTGTA					
*HMBS*	XM_005689536.3	F: GCAACGGCGGAAGAAGACA	59.4	267	−3.316	100.3%	0.994
		R: CAGCGAGTGAACAACCAGG					
*TOP2A*	XM_005693780.3	F: AGCCCATTGGTCAGTTTGGT	55.0	218	-	-	-	
		R: ACCAATTCCTTCAGCGCCAT						
*IGF1*	XM_005680537.3	F: CAGTCACATCCTCCTCGCAT	61.3	112	-	-	-	
		R: AGAGCATCCACCAACTCAGC						

R^2^ refers to the correlation coefficient. Generally, R^2^ > 0.98 is considered feasible and linear by default.

**Table 2:** Expression stability of RGs according to Bestkeeper analysis.

Gene Symbol	SD	CV	r	Rank Order
*RPS4X*	0.33	1.79	0.973	1
*RPS6*	0.38	2.15	0.943	2
*DYNLL1*	0.36	1.71	0.930	3
*RPL7*	0.31	1.75	0.917	4
*RPS20*	0.36	2.05	0.916	5
*RPL3*	0.37	2.03	0.914	6
*RPS26*	0.35	1.99	0.889	7
*RPS27A*	0.26	1.49	0.872	8
*YWAHZ*	0.33	1.58	0.794	9
*KARS*	0.32	1.46	0.730	10

standard deviation (SD); coefficient of variance (CV); correlation coefficient (r).

## Data Availability

Data sharing is not applicable to this article.

## Supplementary Materials

The following are available online at https://www.mdpi.com/article/10.3390/ani11113137/s1, Table S1: The stably expressed genes identified in the goat rumens by RNA-sequencing, Table S2: RNA quality of the samples used in this study, Figure S1: Agarose gel electrophoresis for the candidate reference genes, Figure S2: Gradient dissolution curves of reference genes, Figure S3: Standard curves of reference genes, Figure S4: The relative expression of *IGF1* and *TOP2A* normalized to the different RGs. * *p* < 0.05, ** *p* < 0.01.
