# Supplementary material for: Genome-Wide Identification of Reference Genes for Reverse-Transcription Quantitative PCR in Goat Rumen
Source: Animals (Basel). 2021 Nov 2;11(11):3137. doi: 10.3390/ani11113137 (PMC8614340; doi:10.3390/ani11113137)
Supplement: Supplementary file 1 [file animals-11-03137-s001.zip › Table S2.pdf]

Table S2: RNA quality of the samples used in this study.

Sample ID	Age	28S/18S	OD260/280	RIN value
1（080）	2-month-old	2.08	2.22	8.10
2（082）	2-month-old	2.47	2.05	8.50
3（090）	2-month-old	2.29	2.10	8.10
4（107）	2-month-old	1.64	2.05	8.30
5（157）	2-month-old	2.2	2.06	7.90
6（174）	2-month-old	2.07	1.87	8.00
7（14）	1-year-old	2.06	2.12	8.50
8（15）	1-year-old	2.06	2.24	8.70
9（16）	1-year-old	2.08	2.21	8.20
10（17）	1-year-old	2.07	2.23	8.50
11（18）	1-year-old	2.06	2.11	8.20
12（19）	1-year-old	2.08	1.95	8.90
