# Supplementary figures and images for: Genome-Wide Identification of Reference Genes for Reverse-Transcription Quantitative PCR in Goat Rumen
Source: Animals (Basel). 2021 Nov 2;11(11):3137. doi: 10.3390/ani11113137 (PMC8614340; doi:10.3390/ani11113137)

## Slide 1
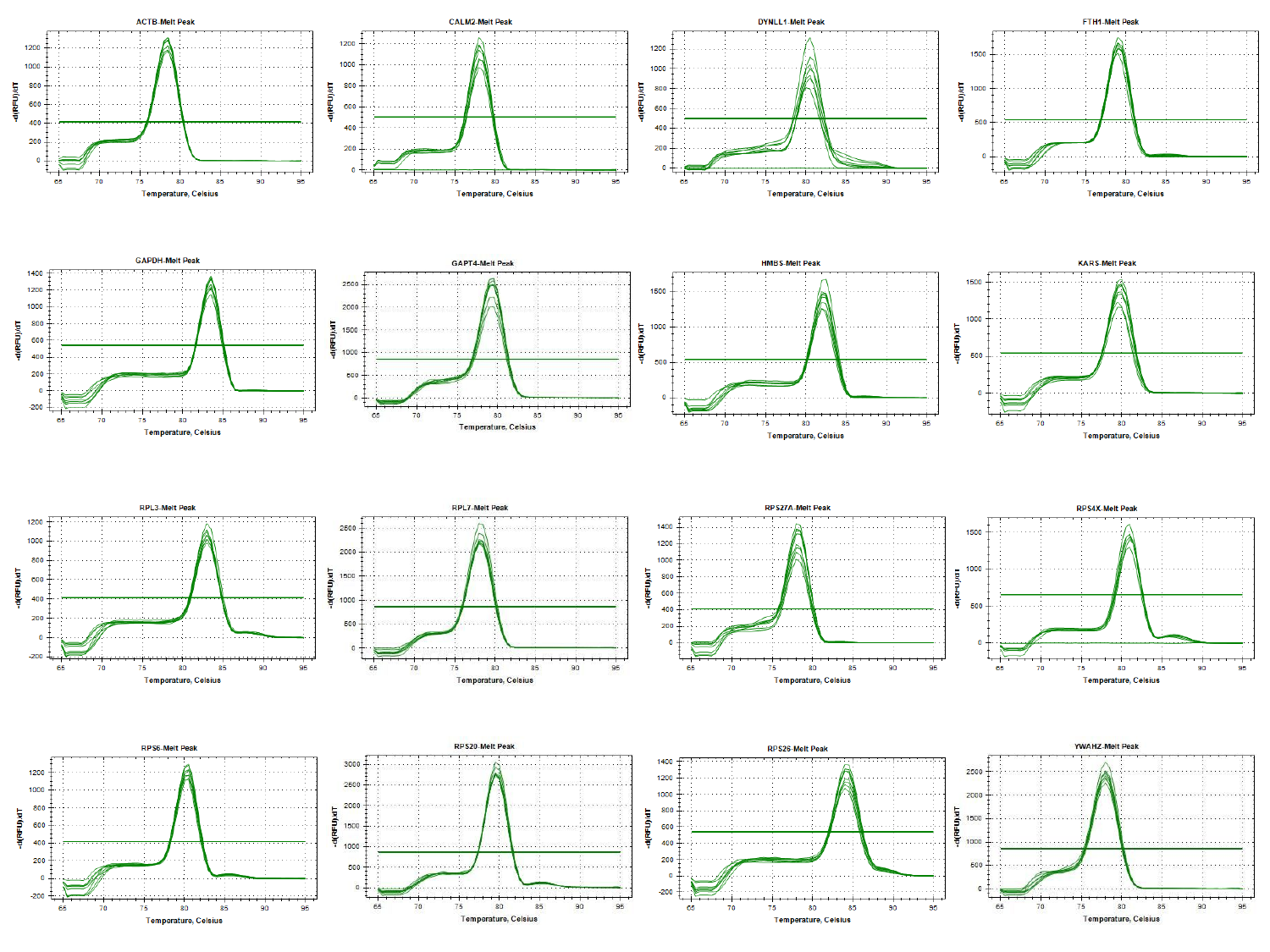

Supplement: Supplementary file 1 [file animals-11-03137-s001.zip › Figure S2.ppt]
